# Varied residential options for gestating sows ensured welfare and productivity

**DOI:** 10.1186/s40813-025-00477-y

**Published:** 2025-12-04

**Authors:** Per Wallgren, Sven-Erik Johansson, Mikael Kirk, PerArne Mattsson, Lena Lindahl, Mate Zoric

**Affiliations:** 1 Department of Animal Health and Antimicrobial Strategies, Swedish Veterinary Agency, Uppsala, SE-751 89 Sweden; 2 Department of Clinical Sciences, Faculty of Veterinary Medicine and Animal Sciences, Swedish University of Agricultural Sciences, Uppsala, SE-750 07 Sweden; 3 Nibble Lantbruk Ltd., Västerås, 725 95 Sweden; 4 Agrisys A/S, Cypresvej 3, Herning, 7400 Denmark; 5 Agritekt M & P Ltd., Brogatan 31, Heby, 744 32 Sweden

**Keywords:** Animal welfare, Behaviour, Electronic sow feeding, Motion, Transponder technology

## Abstract

**Background:**

Adult sows spend more than 50% of their time in units for gestating sows. Consequently, the functionality of these facilities is important for their well-being. This project aimed to depict the well-being of loose housed gestating sows in uninsulated buildings by documenting behaviours, aggressions, stereotypical behaviours and treatments/cullings of sows. Sows were fed individually with an animal-adapted transponder technology with four eating cubicles that allowed individual feeding adjustments. Between meals, the sows could choose from occupying themselves in a tent with deep litter straw or a barn with sawdust and chopped straw.

**Results:**

Sows weaned 13.0 ± 0.7 piglets per litter at a mean age of 31 days with a mean weight of 11.2 ± 0.9 kg. Everyday monitoring and handling of 150–160 gestating sows demanded 32 ± 12 minutes per day of the staff. Medical treatments (9%) and cullings (1%) of non-lactating sows were rare. During gestation, most of the sows preferred to stay in the tent where they mainly rested, but daytime there were always sows in the barn where they were more active. Air temperatures and humidity remained comfortable throughout the year but differed between seasons. During spring, summer and autumn, queuing in front of the eating cubicles were rare and few aggressions were recorded. The activity of the sows was lowest during summer when sows also rested individually. Sows were most active during winter when they rested and moved in groups. Consequently, queuing and interactions in front of the feeding cubicles increased. However, most of these interactions were directed to the side or rear of another sow, and not against the head. Sows ate hierarchically, old sows ate first during the day and gilts last. No stereotypic behaviour was recorded.

**Conclusions:**

Providing sows with varied residential options between meals and individually adjusted feeding during the gestating period ensured motion and well-being without reducing productivity and with low incidences of aggressions and medical treatments/cullings. The study also confirmed that gestating sows can be housed in uninsulated buildings during winter, if they are shielded from wind and can guard themselves from chill, e.g. by bivouacking themselves in deep litter straw.

## Background

For economic reasons, pig farmers have prioritized cost-effectiveness, not the least in building constructions. Gestating sows have often been housed in individual stalls or small groups with a shared manure alley. However, the times they are a-changing and these systems are not considered animal-friendly by the standards of today, although they are still used in parts of the world [1]. In Sweden, free-range systems for gestating sows were mandated by law in 1988 [2], leading to significant improvements in animal welfare [3, 4]. Confinement of sows has also officially been banned within EU since 2013, except for four weeks after service and one week before the expected time of farrowing [5], although not implemented everywhere [6, 7].

Interestingly, sows performed better in a herd when kept in groups of 40 sows on deep litter with a total area of 156 m^2^ corresponding to 3.9 m^2^ per sow than in pens sized 24.5 m^2^ with 8 sows or 10 gilts corresponding to 2.5 to 3.1 m^2^ per animal during the gestation period which indicated improved productivity through increased welfare [8]. Still, there is no scientific consensus regarding the optimal way to house free ranging sows during gestation. Housing larger groups of sows on deep litter straw promote natural behaviours such as rooting and provides a larger total space where lower-ranked individuals can escape dominant sows, which reduce conflicts [9–12]. Still, feeding has remained an issue, because dominant/large sows tend to steal food from low-ranked/small sows [10]. This has been addressed by temporarily confining sows in individual cubicles during feeding, which makes feed stealing impossible but limit automated feeding to a standard ration. Supplementary feeding of thin sows has remained challenging since the eating cubicle used by individual sows vary between days and cannot be predicted by predetermined automated feeding systems [13].

The advancements in transponder technology with small transponders placed in the ears of the sows have improved possibilities for individual feeding rations considerably [14, 15]. However, also the design of the eating cubicles is of importance. With one-way directed eating cubicles, sows are protected by the closed entry to the cubicle when eating and there is no gain for dominant sows to wait at the exit of the eating cubicle [16]. This enables improvements in the designs of the general space for gestating sows with increased opportunities for sows to choose their place of residency when not eating. However, it is crucial to objectively assess whether such innovations truly enhance animal welfare.

The aim of this study was to document welfare by registering, general and stereotypic behaviours, as well as aggressions between sows and to evaluate the functionality of a transponder regulated electronic sow feeding (ESF) system where gestating sows could choose between two different areas with various types of bedding options between meals.

## Materials and methods

### Animals and buildings

The study was conducted in a Swedish farrow to finish farm with 280 conventional Yorkshire ×Landrace sows mated with Duroc semen. The reproductive cycle of sows in the herd was 152 days. Of these, they spent 70 days in the farrowing and mating units. Thus, 82 days (54% of the time) were spent in the building for gestating sows.

**Fig. 1:**
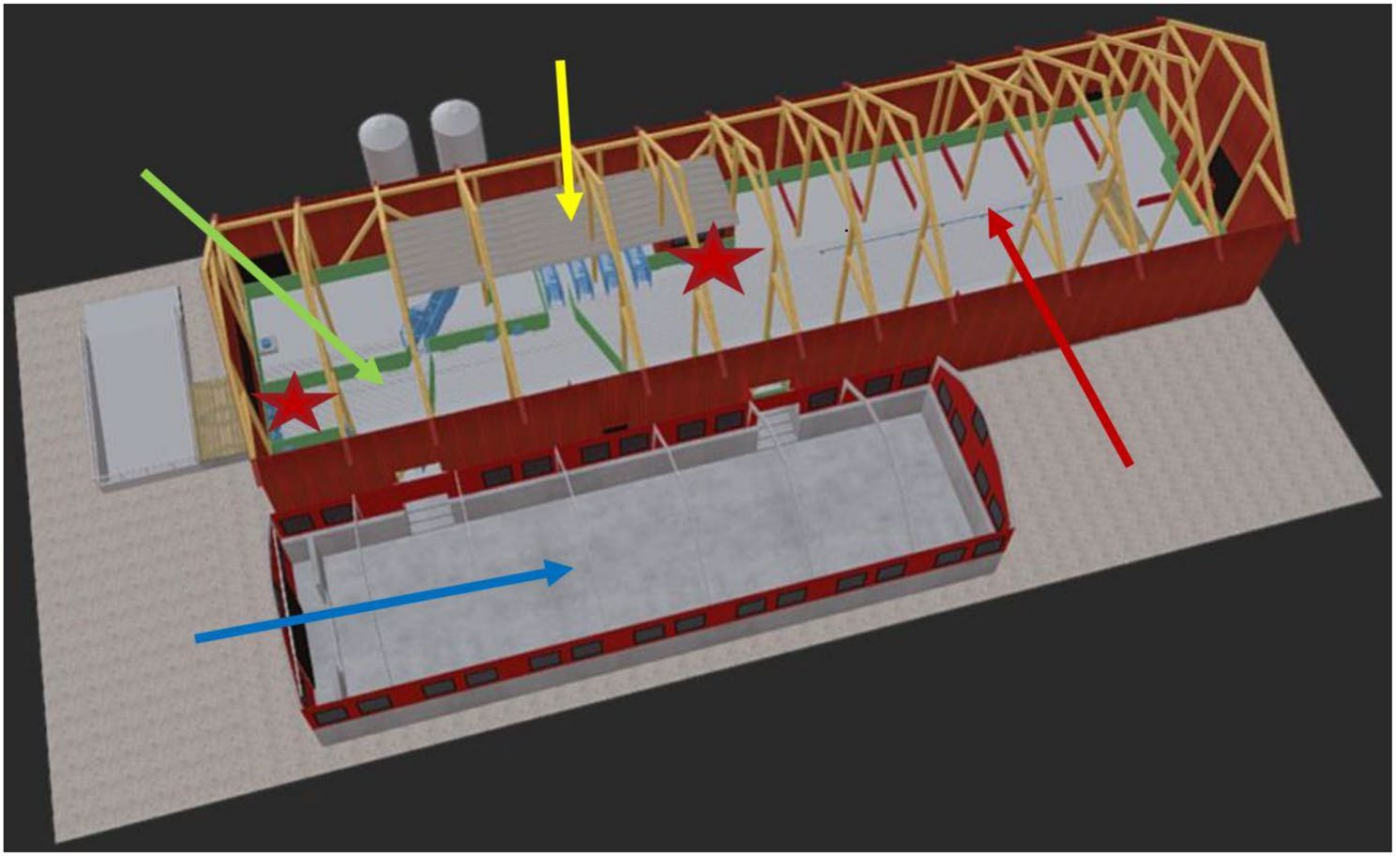
The facilities for gestating sows. Sows enter the one way directed eating cubicles (*n* = 4, yellow arrow) from the pre-eating area and leave them at the post eating area (green arrow). From the post-eating area, the sows have access to the tent with deep litter straw (blue arrow). From the tent sows move forward to the barn with chopped straw (red arrow), from where they again can reach the pre-eating area. Water throughs are indicated by red stars

A barn sized 518 m^2^ (43.2 × 12 meters), was rebuilt for a transponder system with four eating cubicles (Freeda, Agrisys A/S, Herning, Denmark). The entry for the staff to the barn with a visual view over the feeding cubicles and the technical control station altogether covered 13 m^2^. The remaining 505 m^2^ of the barn was disposed by gestating sows (Fig. 1).

**Fig. 2:**
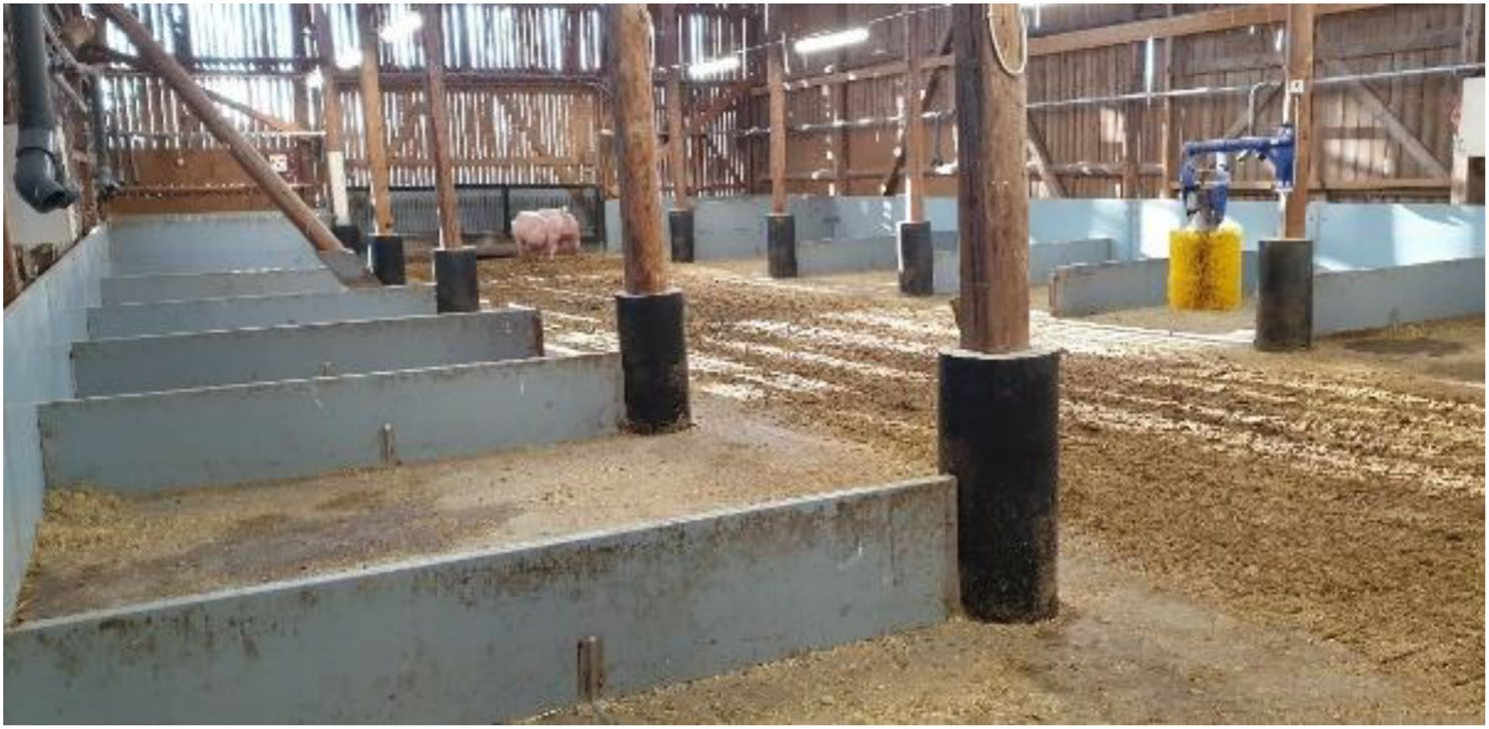
The barn with 14 wind-sheltered pens with saw dust and chopped straw and the brush for scratching (Camera 4)

**Fig. 3:**
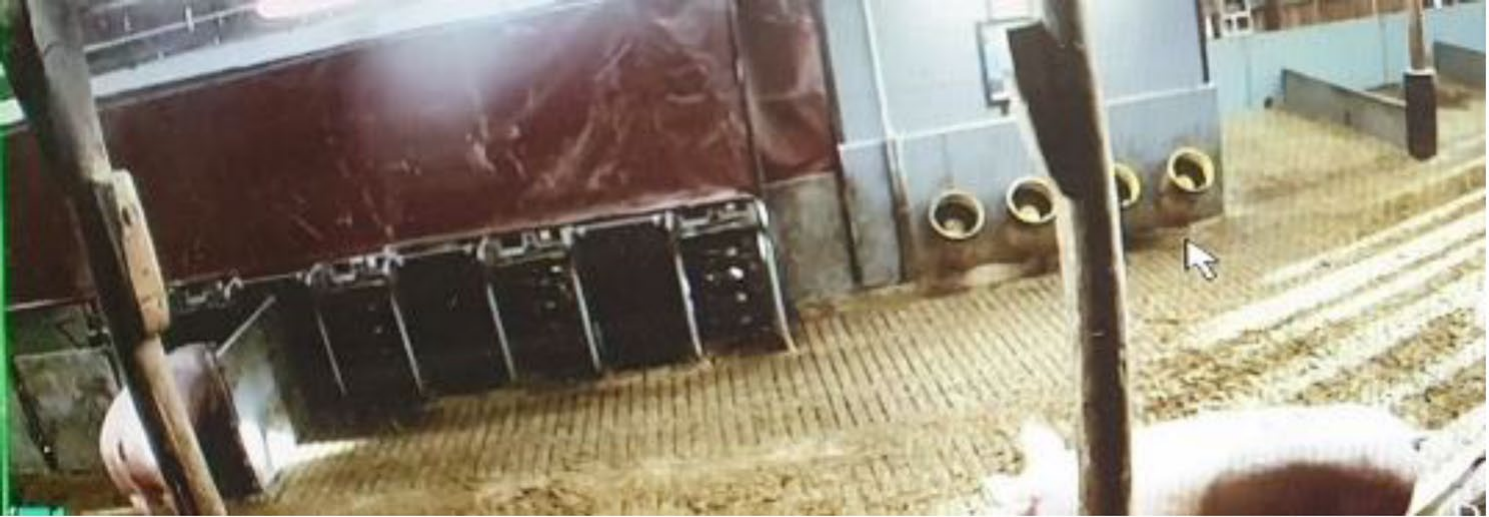
The pre-eating area with entrance to four feeding cubicles and four water troughs (Camera 1)

**Fig. 4:**
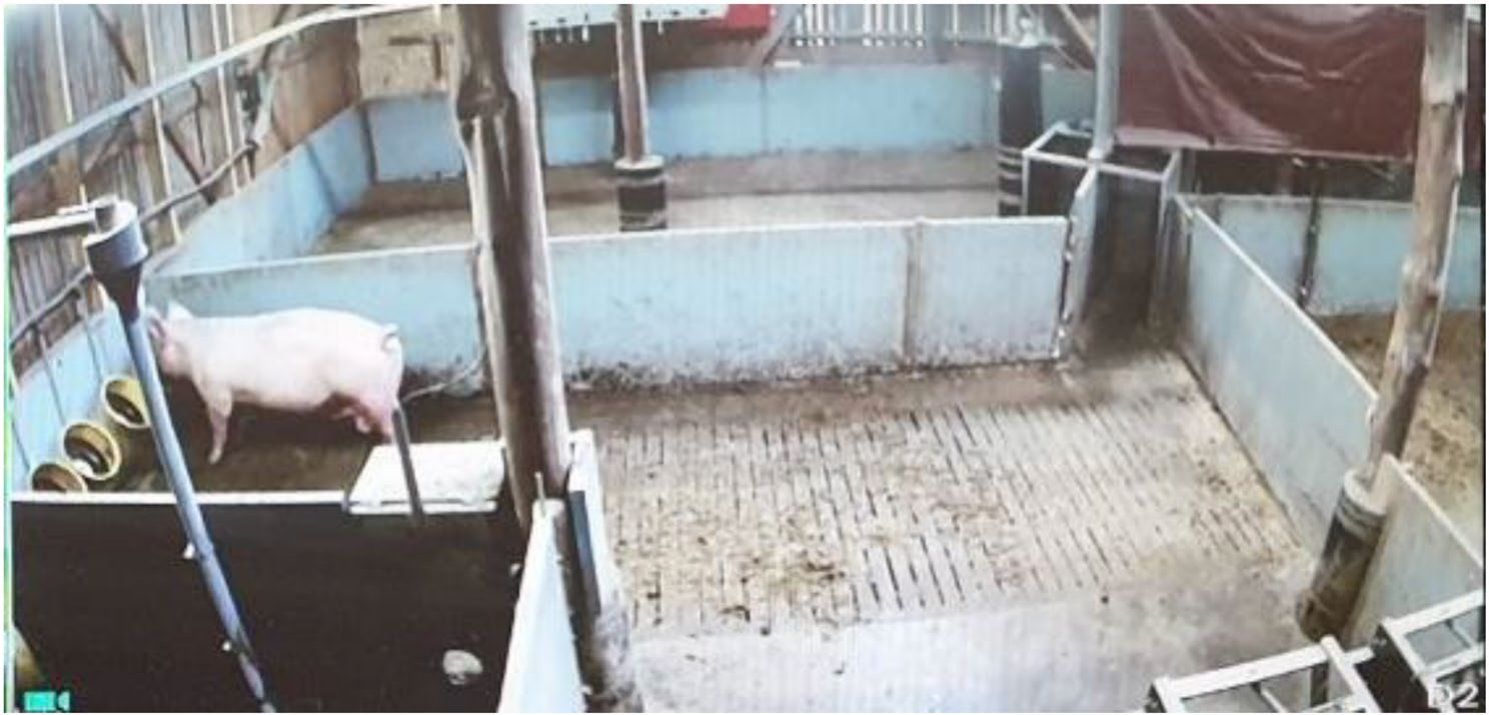
The post-eating area with four water troughs and the pen for handling sows in the rear (Camera 2)

In the barn, gestating sows had free access to a recreation area of 254 m^2^ (21.2 × 12 meters), where an aisle was surrounded by 14 pens (potential laying areas). The saw dust spread in the laying areas was complemented with chopped straw. As the main aim of the straw was recreation/occupation of sows, smaller amounts of straw were assigned daily because pigs only find straw attractive when they regard it as new/fresh [17]. This area also housed an automatic scrubbing machine for cows (SCB, DeLaval, Tumba, Sweden) and a shower automatically sprinkling for one minute per hour at temperature above 21 °C (Fig. 2). The remaining area of the barn with four eating cubicles (114 m^2^) had a pre-eating area sized 50 m^2^, (Fig. 3), a post-eating area (30 m^2^) with access to a boar in a pen (10 m^2^) with the aim to identify sows returning to oestrus, a collection area of 40 m^2^ for sows to be handled, training areas for gilts (32 m^2^) and pens (15 m^2^) for potentially diseased/injured sows (Fig. 4).

**Fig. 5:**
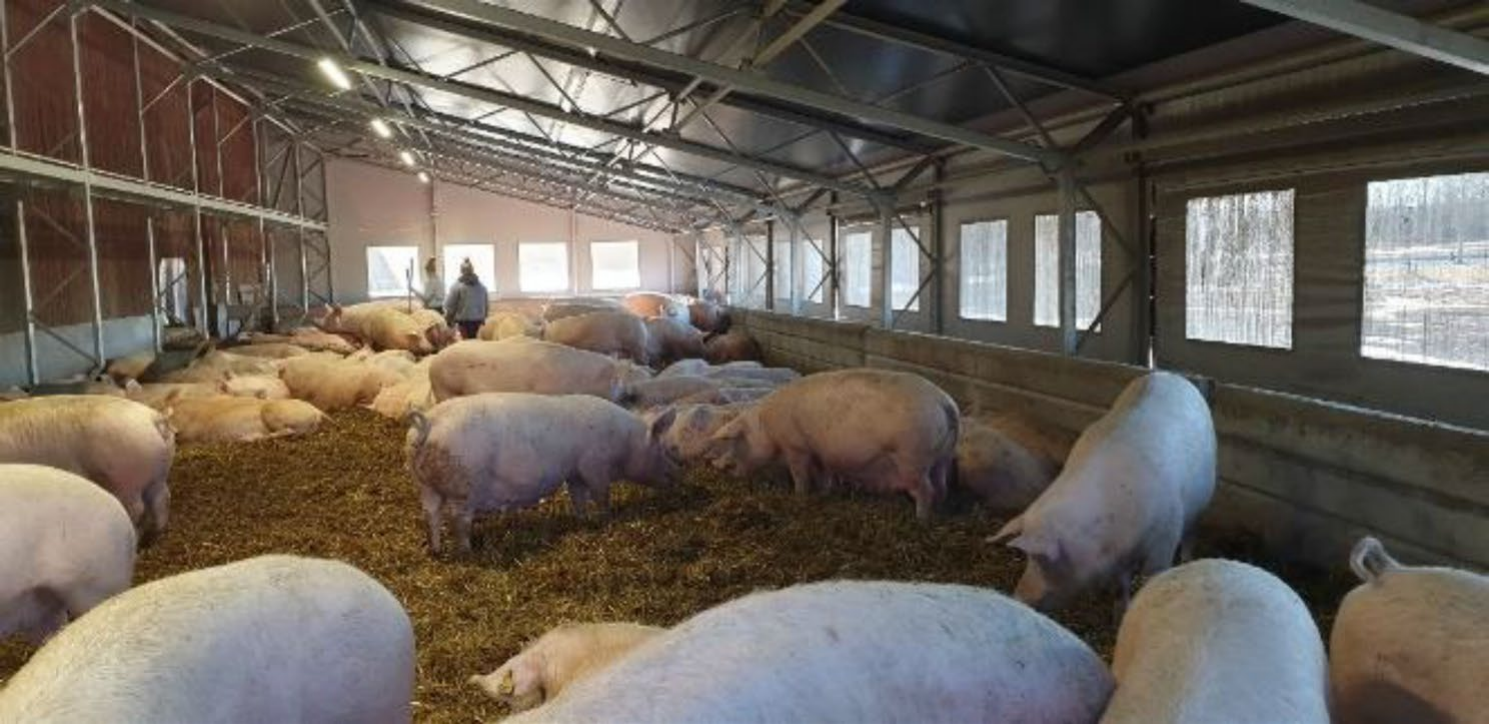
The windowed tent with deep litter straw (Camera 3)

The post-eating area and the recreation area in the barn were connected by a resting area in a tent of 200 m^2^ (25.8 × 7.72 meters). The tent, with plastic windows were built on a concrete slab and the floor was covered with deep litter straw (Fig. 5).

Thus, the sows disposed 705 m^2^, corresponding to 4.4 m^2^ per sow. When not eating, sows had access to 534 m^2^ (the recreation area of the barn, the deep litter straw bed in the tent and the pre- and post-eating areas) which corresponded to an area of 3.4 m^2^ per sow.

### Functionality of the buildings

Figure 1 shows the housing system for gestating sows that was developed at Nibble Lantbruk Ltd, Västerås, Sweden, in collaboration with Agrisys A/S, Herning, Denmark and Agritekt M &P Ltd, Heby, Sweden, and in consultation with the Swedish Veterinary Agency (SVA), Uppsala, Sweden.

In the pre-eating area sows could choose when to enter and eat at one of the four feeding cubicles, corresponding to one eating station per 40 sows, located just to the right of the two feed silos at the far side of the main building (Fig. 1, yellow arrow and Fig. 3). The transponder system allowed individually adjusted feeding. The sows were automatically weighed before feeding and the feed portion was automatically adjusted according to the need (weight) of each sow.

The feeding day started at 03.00 and each sow had a defined ration of food per day, depending on sow size, which was distributed in doses of 88 or 104 gram dry flour feed depending on feed type. The flour was served as soon as the head of a sow entered the feeding through of the eating cubicle and it was on average repeated every 20^th^ second, depending on the feed curve of the individual sow/gilt, until the sow left the through. Thus, the sows could consume the whole daily ration during the first visit or divide eating to several feedings. Regardless, no more food was distributed to sows that had consumed their daily ration until the next feeding day and consecutive visits to the feeding cubicles were not awarded. At decreasing sow body weights, the system alarmed and increased the feed rations above the norm to affected sows.

When leaving the feeding station, sows were automatically directed to the post-eating area or to the pen for controlling/handling sows (Fig. 1, green arrow and Fig. 4). Sows could not re-enter to the one-way-directed feeding cubicles. In the post-eating area, sows passed a boar with the aim to detect sows that had returned to heat. There were four water dispensers with troughs located at both the pre- and post-eating areas (Fig. 1, red stars and Figs. 3 and 4).

When leaving the post-eating area, the sows proceeded to the side “building”, a tent with plastic windows placed on a concrete slab with a deep litter straw bed where the sows could rest, eat straw and root (Fig. 1, blue arrow and Fig. 5).

The sows could choose to stay in the tent or continue to the barn with 14 potential lying areas with space for several sows (Fig. 1, red arrow and Fig. 2). These stalls were bedded with saw dust and chopped straw (i.e. not deep litter straw). From here, the sows had the option to visit the feeding stations again. They could also return to the tent.

### Productivity and health status

The productivity of the sows was documented during 2024 and defined as piglets produced per litter and their weight at weaning at 31 days of age. Replacement of sows post weaning were documented and defined as planned or undesired due to injuries or non-pregnancy.

The health status during gestation was defined by documenting medical treatments and cullings of gestating sows, as well of sows during lactation/mating. These measures were effectuated and documented by the staff following written instructions by the herd veterinarian.

### Climate recordings

Air temperature and humidity in – and outdoors were recorded automatically (Mini-datalogger testo 174-H NEW, Testo AG, Baden-Würtenberg, Germany), every hour continuously throughout the study. The Temperature-Humidity Index (THI) was calculated for each observation day according to the formula:

THI = Temperature (°C) - [0.55 – (0.0055 ×relative humidity (%)] x [Temperature (°C) − 14.5] [18]. A THI value of 23 or higher is considered dangerous for pigs due to heat stress [19] and could also affect the productivity of sows [20].

The meteorological seasons were defined by the Swedish Meteorological and Hydrological Institute [21]. The spring starts during the first out of seven consecutive days with a mean outdoor temperature for the 24 hours of a day ranging between 0 and 10 °C, generally in March. The summer begins during the first out of five consecutive days with a mean outdoor temperature above 10 °C, in general in June. The autumn starts during the first out of five consecutive days with a mean outdoor temperature ranging between 10 and 0 °C, in general in September. The winter starts during the first out of five consecutive days with a mean outdoor temperature below 0 °C, in general in December.

### Video recordings and criteria for filming

To document the behaviour of the sows and the functionality of the facilities for gestating sows, sows were filmed. Four different areas in the buildings for pregnant sows were filmed: the pre- and post-eating areas, the tent and the barn (Figs. 1–5). With the aim to document any differences in sow behaviour due to season, these recordings covered a full year (2024). For each season, one day for filming was randomly selected and filmed for 24 hours. Unfortunately, there were camera errors during the winter filming scheduled for 2024. Therefore, the winter 2024 filming was replaced with filming in winter 2025, again on a randomly selected date.

Monitoring was conducted with Monacor, IOZ-408BV, COMFORT Line video surveillance set, consisting of COMFORT Line 8-channel network video recorder IOR-208 and 4 ×COMFORT Line 3 megapixel network colour camera IOC-2812BV, http://www.monacor-international.com. Each camera captured one frame per second, which amounted to 86,400 frames per camera and day. Each frame was manually analysed according to predefined behaviours in accordance with a previously used model [22, 23]. If behaviours previously not defined were observed, they were to be inserted into that model.

As the sows had constant access to the feeding cubicles, and as previous studies have shown that low-ranked individuals often eat during less attractive hours of the day [17], it was important to cover the whole day. Also filming in presence and absence of caretakers was important, as the presence of humans affects the activity of pigs [17].

### Behaviour

Behaviours and activities of gestating sows were recorded manually according to predefined standards. They were recorded over a 24-hour period per season and summarised as numbers, durations (in minutes), or percentages during the observed 24-hour period.

Behaviours were categorized as either positive (desired), such as non-aggressive interactions between animals and exploratory behaviour, or negative (undesired), such as aggressions and stereotypical behaviours.

The behaviour of sows was classified as:

1. Activity: Standing, walking, or rooting; located at the tent or at the barn or at the entry of these facilities.
2. Resting: Lying, but not sleeping.
3. Sleeping: Lying posture with eyes closed; sows were unresponsive to the environment.
4. Grouping of sows when resting in the tent: Categorized as individual, < 5, 5–10, 10–25 or > 25.
5. Feeding: Number of sows waiting at feeding stations. Queuing was defined as more than two sows awaiting at each feeding cubicle.
6. Drinking: Number of sows drinking at the water throughs at the entry or exit of the feeding stations. Total time spent drinking and total time with at least one sow queuing in front of the water throughs were recorded as minutes per day.
7. Interaction with the boar: Number of sows staying at the pen of the boar with apparent interest of the boar, i.e. possibly indicating sow in heat.
8. Interaction between sows: Number of interactions between sows directed to head, body or rear, and where these interactions took place (i.e. pre- or post-eating areas, tent or barn). Interactions head-to-head were defined as interactions/aggressions for social hierarchy, whereas interactions to body or rear were defined as non-aggressive interactions aimed to create space.
9. Stereotypic behaviours. Behaviours that were repetitive, identical and possessed no obvious goal or function. Primarily chewing on furniture or playing with ear/tails of other sows, but we searched for any type of stereotypical behaviour repeated more than twice.

### Eating order

During the study, a question of eating order among sows was raised. Therefore, all visits to the feeding cubicles were recorded during 1^st^ to 5^th^ of April 2025 (spring) and assorted according to parity number of the sows. Visits to the feeding cubicles were recorded individually and included time for the visit and cubicle visited. We did not have the technical options to differ between successful and unawarded visits to the cubicles, but as the feeding day started at 03.00 visits made between 00.00 and 02.30 were defined to be unawarded.

Sows turn heavier during the end of the gestation which potentially could change their eating habits in a system were sows decided individually when to eat. Did they defend their position, or did they eat when the general interest of the feeding cubicles was low? To study whether sows changed their behaviour during the end of the gestation, all visits to the feeding cubicles were documented for 25 sows four days before expected farrowing (sows were commonly transferred to the farrowing pen three days before expected farrowing).

### Activities of the staff

The staff was also captured by the cameras and their presence in the sow facilities (but not in the control room) was recorded manually during the filmed days. The activity of the staff was recorded as presence in the building not differentiating between activities such as observing, handling or treating sows.

### Statistics

The main aim of this study was to document the system with varied residential options for gestating sows from functional and welfare point of views. Descriptive statistics were used to summarise the main findings regarding the functionality and welfare outcomes of the housing system. For a national comparison, the mean productivity and health status (medical treatments and cullings) of Swedish sows affiliated to PigVision® (AgroVision, Apeldoorn, The Netherlands) is also presented in results.

Appropriate statistical methods were applied to the comparison of behavioral and health parameters of gestating sows across different seasons. Qualitative traits (such as the presence or absence of specific behaviours) were analysed using chi-squared tests (χ^2^-tests), while quantitative traits (such as mean activity or health indicator values) were compared using t-tests by groupwise comparisons (one degree of freedom). The specific statistical method used for each comparison is indicated in parentheses in Results. All analyses were conducted using complete observation datasets from the excel spread sheets in which the results were documented.

## Results

### Productivity

In mean, sows gave birth to 15.0 ± 0.6 live born piglets per litter and weaned 13.0 ± 0.7 piglets at 31 days of age with a mean weaning weight of 11.2 ± 0.9 kg during 2024.

The corresponding mean figures for Swedish herds affiliated to PigVision® (AgroVision) were 15.6 live born and 13.1 weaned piglets at an age of 32.4 days and a weight of 9.0 kg per sow and year [24].

### Health status and medical treatments

There were no alarms due to decreasing body weights of sows during the stay in the building for gestation sows. During 2024, there were 25 antibiotic treatments effectuated in non-lactating sows (12 in the unit for gestation sows and 13 in the mating unit). This corresponded to an annual treatment incidence of 9% of the sows of the herd during the non-lactating period. Of these, 22 were due to lameness, corresponding to an annual treatment incidence of 8% of the sows for lameness. During suckling, a total number of 96 sows (34%) were treated with antibiotics; 49 due to mastitis and 41 due to post partum dysgalactia syndrome (PPDS, of which 12 occurred in one farrowing batch in May), 4 due to lameness. and 2 for other reasons.

The report on annual antibiotic treatments of sows in the Swedish herds affiliated to PigVision® (AgroVision) for one year did not differ between treatments effectuated during lactation and gestation. In total, 50% of the sows were treated with antibiotics during the year; 23% due to lameness (arthritis of claw lesions), 13% due to mastitis and 8% due to PPDS [24]. When all treatments were merged for the sows in the ESF system with varied residential options during gestation, they corresponded to 42% of the sows being treated, 9% due to lameness, 18% due to mastitis and 15% due to PPDS.

The incidence of medical treatments did not differ between the categories (*p* > 0.05, χ^2^-test), but the incidence of treatments due to lameness was lower in the ESF system with varied residential options for gestating sows (*p* < 0.01, χ^2^-test).

### Culling of sows

Three of the 22 sows treated for lameness did not respond to treatment and waere culled for welfare reasons, corresponding to a culling incidence of 1% in the ESF system. The culling incidence of sows in the herds affiliated to PigVision® (AgroVision) was 8% (3,5% due to locomotion disorders, 1.5% due to poor condition, 0.5% due to trauma and 2.5% miscellaneous) [25]. The difference in incidence of culling sows was significant (*p* < 0.05, χ^2^-test).

### Replacement of sows

During 2024, 95 sows were replaced post weaning (34%). Of these, 65% were planned due to age (*n* = 39, 41% of all replacements), poor production/unmanageableness (*n* = 14, 15% of all replacements) and udder deficiencies (*n* = 6, 6% of all replacements). The non-planned replacing was dominated by non-pregnancy (*n* = 17, 18% of all replacements). The remaining 19 sows were replaced for various other reasons. When the three sows that were culled during gestation were included, the overall incidence of replaced sows was 38% (98 out of 280).

In mean, sows in the Swedish herds affiliated to PigVision® (AgroVision) were replaced after 4.0 ± 2.2 parities [25]. Of the annual replacements of 26%, 11% were due to reproductive disorders, 6% due to udder deficiencies and 4% of the replacements were planned due to production or age [25]. However, as described above another 8% of the sows were culled. Thus, the overall incidence of replaced sows was 34% [25].

The extent of the replacement did not differ between the categories (*p* > 0.05, χ^2^-test), but replacements were to a larger extent planned due to production qualities in the ESF system with varied residential options for gestating sows (*p* < 0.001, χ^2^-test).

### Climate recordings

The temperature and air humidity differed between seasons but also varied within individual days (Table 1). However, the THI-index never exceeded 23.

**Table 1:** Mean values and range of climatic factors registered

	Outdoors	Tent	Barn
	Mean ± SD (range)	Mean ± SD (range)	Mean ± SD (range)
Spring Outdoor temp 0 to10°C*			
Temperature (˚C)	7.4 ± 4.7 (1–13)	10.7 ± 4.9 (4–17)	8.8 ± 3.2 (4–13)
Air humidity (%)	54 ± 15 (32–75)	51 ± 13 (34–67	55 ± 13 44 -66)
**THI-index of the mean**	**9**	**12**	**10**
Summer Outdoor temp > 10°C*			
Temperature (˚C)	17.1 ± 4.8 (10–23)	20.6 ± 5.1 (13–27)	18.2 ± 3,9 (13–25)
Air humidity (%)	44 ± 8 (33–58)	44 ± 9 (30–57)	43 ± 8 (29–56)
**THI-index of the mean**	**16**	**19**	**17**
Autumn Outdoor temp 10 to 0°C*			
Temperature (˚C)	2.1 ± 0.6 (1–3)	7.1 ± 0.4 (6–8)	6.0 ± 0.5 (5–7)
Air humidity (%)	89 ± 3	89 ± 1 (87–90)	78 ± 1 (76–81
**THI-index of the mean**	**3**	**8**	**7**
Winter Outdoor temp < 0°C*			
Temperature (˚C)	−16.7 ± 1.5 (−19 to −14)	−7.4 ± 1.2 (−9 to −5)	−10.2 ± 2.3 (−13 to −6)
Air humidity (%)	100	94 ±1 (92–97)	79 ± 5 (68–85
**THI-index of the mean**	**−17**	**−7**	**−7**

THI-index = Temperature Humidity index. * = mean temperature of the 24 hours of the day

### General behaviour. Camera 1, the pre-eating area

**Fig. 6:**
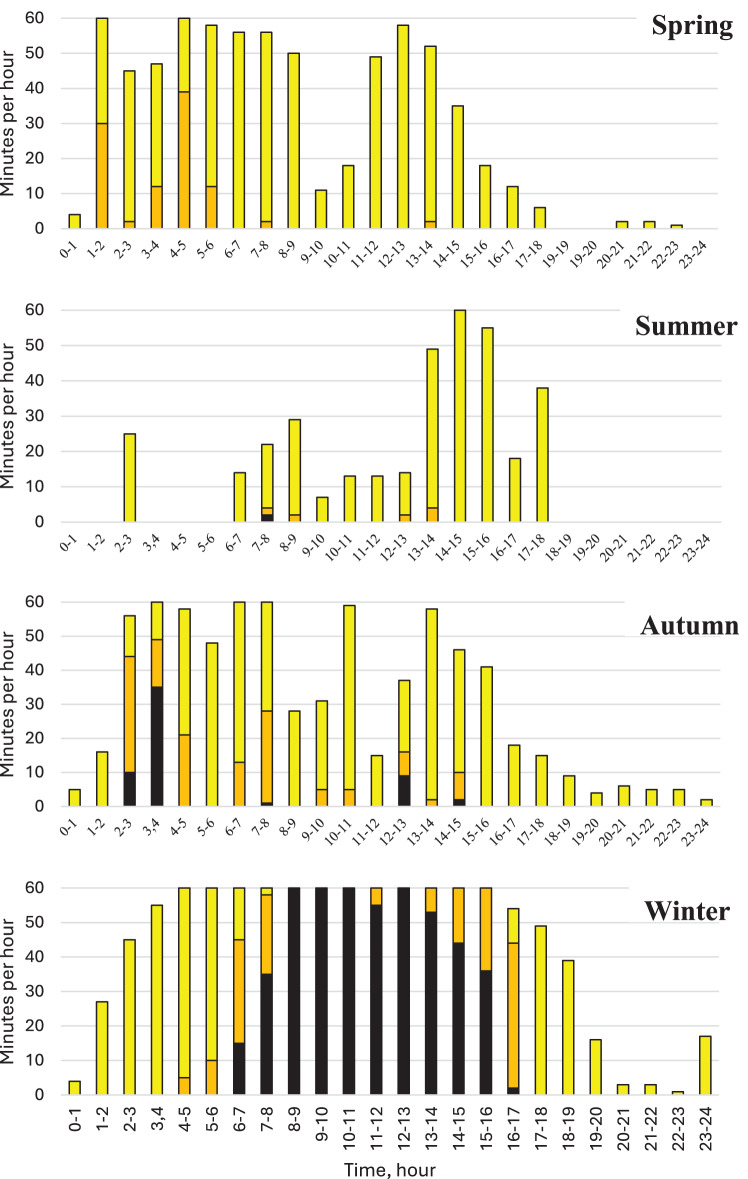
Number of sows waiting at the feeding stations during the day. Maximum one sow per feeding station (yellow), maximum two sows per feeding station (brown) or queuing which was defined as more than two sows per feeding station (black). There were significantly more sows queuing during winter than during the other seasons (*p* < 0.001, t-tests)

Each sow had a fixed portion of feed per 24 hours, which could be consumed from 03.00 am and during the subsequent 24 hours. The number of sows waiting at the entrance to the feeding cubicles are shown in Fig. 6. During spring and autumn, sows approached the feeding stations prior to the beginning of the feeding day and forenoon, but rarely during the late afternoon and evening. Queuing, defined as more than two sows awaiting at each feeding cubicle, was not monitored during spring, for 2 minutes during summer, and for 55 minutes during autumn. In summertime, sows ate later during the day than during the other seasons and they ate in absence of queuing with exception for two minutes during the whole day. In contrast, sows gathered at the pre-eating area during winter, and during daytime more than two sows awaited at the feeding stations for 480 minutes, which differed from the other seasons (*p* < 0.001, t-tests).

Sows visited the eating cubicles and drinking throughs all over the day, but to a lesser extent in the evenings when the ration of the day was consumed. In general, there was no queuing at the water throughs with exception of wintertime when the total time with at least one sow waiting at the throughs at the pre-eating area was 200 minutes during the day, concentrated to daytime (*p* < 0.01 to the other seasons, t-tests). During spring, summer and autumn, that figure was 10, 1 and 0 minutes, respectively.

### General behaviour. Camera 2, the post-eating area

The sows mainly used this area as a passage for the transfer to the tent or to the barn. The boar pen was empty during the filming in spring. The total number of interactions with the boar were 3, 20 and 0 during summer, autumn and winter, respectively. The total time with at least one sow waiting at the water throughs was 17, 1, 0 and 0 minutes during spring, summer, autumn and winter, respectively.

### General behaviour. Camera 3, the tent

**Fig. 7:**
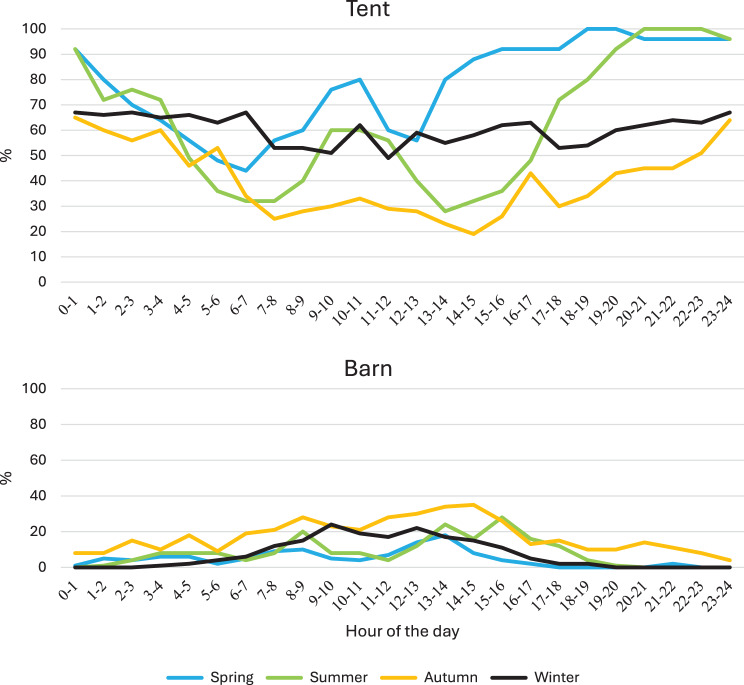
The percentage of sows occupying themselves in the tent or the barn during the day over seasons. During all seasons, there were always more sows in the tent than in the barn (*p* < 0.001, t-tests)

The percentages of the sows that were in the tent with deep litter straw are shown in Fig. 7. There was never crowding ( > 5 sows) at the entrance or the exit to the tent. These spots were completely free from sows for 15 hours during spring, 14.5 hours during summer, 12.5 hours during autumn. During winter, these spots were completely free from sows for 6.5 hours, which differed significantly (*p* < 0.05, χ^2^-tests) from spring and summer.

**Fig. 8:**
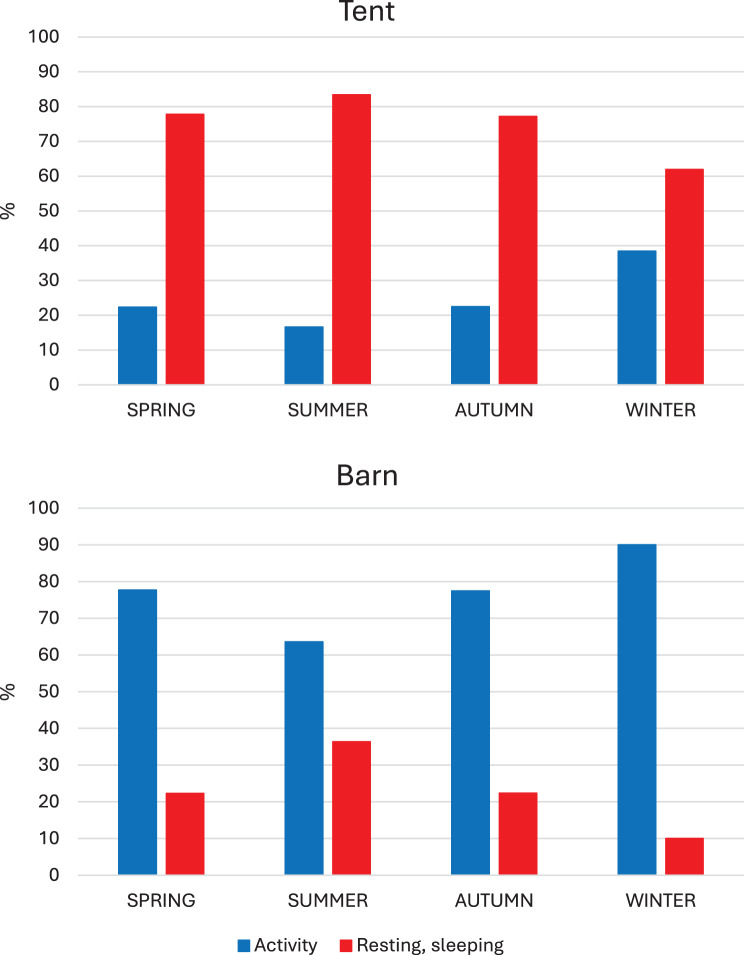
Behaviour of sows in the tent and barn, respectively. Red columns represent resting or sleeping. Blue columns represent activity (standing, moving or rooting). The behaviour differed between the buildings during all seasons (*p* > 0.001, χ^2^-tests)

**Fig. 9:**
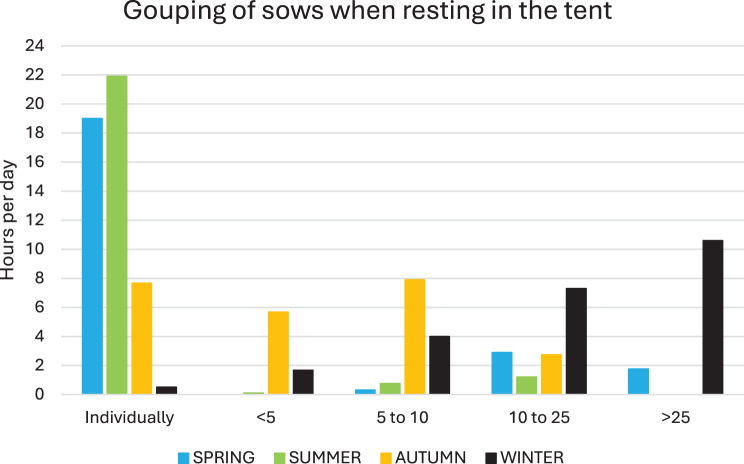
Grouping of sows when resting in the tent depending on season. The group sizes were defined with the aim to visualise whether sows preferred to rest individually, or in small or larger groups

The activities of the sows in the tent were dominated by resting/sleeping (Fig. 8), which covered around 85% of the time during summer and around 60% during winter). As seen in Fig. 9, sows rested groupwise during winter, which differed to the other seasons, especially the summer, when sows rested individually (*p* < 0.001, χ^2^-tests).

### General behaviour. Camera 4, the barn

The percentage of the sows that were in the barn is shown in Fig. 7. As stated above, there was no crowding at the entrance to the barn. In contrast to the tent (*p* < 0.001, χ^2^-tests), the behaviours of the sows were dominated by activities in the barn (Fig. 8).

### Interactions between sows

During spring, one interaction between sows was recorded. It took place at the pre-eating area and was directed head-to-head, which indicated aggression for social hierarchy. During summer, 21 interactions were recorded, whereof 18 took place at the pre-eating area and three in the post-eating area. Of these, 12 were directed to the head, and 6 to other parts of the bodies (5 to the body and 1 to the rear) not indicating interaction associated with ranking. During autumn, 7 interactions were recorded, all at the pre-eating area, 4 directed to the head, 2 to the body and 1 to the rear. During winter, 42 interactions were recorded, whereof 26 took place at the pre-eating area and 15 at the water throughs there. Of these, 12 were directed to the head, 21 to the body and 9 to the rear. Thus, the overall incidence of aggressions (interaction head-to-head) was 7.3 ± 5.6 times a day which corresponded to 0.05 ± 0.04 aggressions per sow and day. Both the total number of interactions and the aggressive interactions were higher during summer and winter than during spring and autumn (*p* < 0.001, χ^2^-tests), The total number of interactions during winter was higher than during summer (*p* < 0.01, χ^2^-test), but the number of aggressive interactions head-to-head did not differ between these seasons (*p* > 0.05, χ^2^-test). Over the year, the mean incidence of interactions between sows were concluded as low; interactions head-to-head were 0.05 ± 0.04 per sow and day, and interactions to body or rear were 0.06 ± 0.08 per sow and day.

### Stereotypic behaviours

Stereotypic behaviours were not recorded during the days of filming.

### Eating order

There were no regroupings of sows when the eating order was scrutinised for five consecutive days (1^st^ to 5^th^ of April 2025). The parity number of the sows, as well as their performance during the preceding farrowing is shown in Table 2. The largest litters were born by 6-parity sows compared to other parities (*p* < 0.05 to 0.001, t-tests). In contrast, 3- and 5-parity sows had the numerically largest numbers of piglets weaned per litter. First parity sows gave birth to smaller litters than 3-, 5- and 6-parity sows (*p* < 0.05 to 0.001, t-tests) and weaned fewer piglets than 2-, 3- and 5-parity sows (*p* < 0.05 to 0.01, t-tests).

**Table 2:** Parity number and productivity during the preceding farrowing of gestating sows in the facilities for gestating sows during 1st to 5th of April 2025. Six-parity sows had more liveborn piglets than all other parities (*p* < 0.05 to 0.001, t-tests). Parity 1 sows had fewer liveborn and weaned piglets than parity 3 and parity 5 sows (*p* < 0.05, t-tests)

Parity number	Number and percentage		Performance, preceding litter		Feeding cubicles, activities per sow and day		
	(n)	(%)	Live born	Weaned	Visits	Stations visited	Too early visits*
**Gilts**	17	13.6	-	-	**3.3 ± 0.7**	**2.4 ± 0.7**	**0.1 ± 0.2**
**1**	25	20.0	**13.6 ± 3.8**	**11.9 ± 2.7**	5.5 ± 0.3	2–9 ± 0.4	**0.1 ± 0.1**
**2**	17	13.6	15.5 ± 2.3	13.3 ± 1.5	**7.4 ± 1.3**	3.3 ± 0–1	0.2 ± 0.1
**3**	19	15,2	15.7 ± 2.5	**13.7 ± 1.2**	6.3 ± 0.7	3.3 ± 0.1	0.2 ± 0.1
**4**	12	9.6	14.7 ± 1.7	12.9 ± 1.5	**7.0 ± 0.4**	3.1 ± 0.6	0.2 ± 0.2
**5**	8	5.6	16.,4 ± 2.1	**13.6 ± 1.1**	5.7 ± 0.8	3.3 ± 0.2	**0.6 ± 0.2**
**6**	8	6.4	**17.9 ± 1.9**	12.3 ± 1.5	5.3 ± 0.4	**3.4 ± 0.1**	0.4 ± 0.2
**7**	10	8,0	14.7 ± 1.7	12.4 ± 2.1	4.9 ± 0.5	3.0 ± 0.2	0.2 ± 0.2
**8–12**	10	8.0	12.6 ± 3.0	**11.8 ± 2.5**	5.1 ±1.0	3.1 ± 0.3	0.5 ± 0.3
**TOTAL**	**125**	**100**	**14.9 ± 3.1**	**12.7 ± 2.0**	**5.5 ±0.2**	**3.1 ± 0.1**	**0.3 ± 0.2**

*The feeding day began at 03.00 am. Too early visits represent visits from 00.00 to 02.30, i.e. expected unawarded visits without access to food

**Fig. 10:**
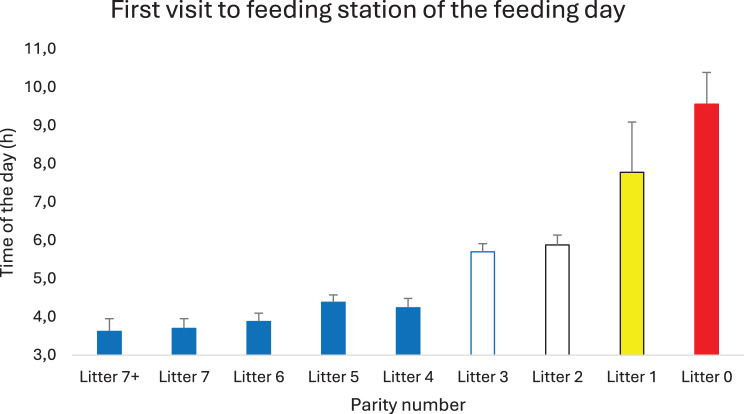
The mean time for the first visit to the feeding cubicles of the day assorted according to parity number. Red bar differs significantly from yellow (*p* < 0.01, t-tests), white (*p* < 0.001) and blue bars (*p* < 0.001). Yellow bar differs significantly from blue (*p* < 0.001) and white (*p* < 0.01) bars. White bars differ significantly from blue bars (*p* < 0.001)

**Fig. 11:**
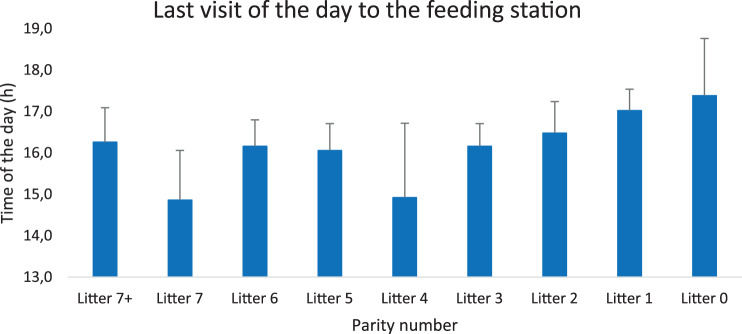
The mean time for the last visit to the feeding cubicles of the day assorted according to parity number. The time for the last visit to the feeding cubicles of the day did not differ between age categories

As evident from Fig. 6 and Table 2, there were few visits to the feeding cubicles from midnight and until the feeding day started at 03.00 am. In mean, the sows visited the feeding cubicles 5.5 ± 2.0 times per day, which required a walking distance of at least 550 meters, and they varied between cubicles. Gilts differed from sows by visiting the cubicles less frequently (3.3±0.7 times per day). As seen in Fig. 10, the first visit to the cubicles after the start of the feeding day at 03.00 followed a hierarchical order where the eldest sows ate first and the gilts last (*p* < 0.001, t-tests). The time for the last visit to the feeding cubicles of the day did not differ between age categories (Fig. 11).

Sows in late pregnancy (4 days before expected farrowing) visited the feeding cubicles 4.0 ± 1.6 times a day (Table 3). Younger sows visited the cubicles later during the day than older sows. The feeding cubicles were visited before four o´clock by sows with parity numbers above 6, while the first parity sows made their first visit to the cubicles at a mean time of quarter past ten.

**Table 3:** Visits to the feeding cubicles during the end of the gestation sows, i.e. four days before expected farrowing

Parity number	(n)	Too early visits*	Visits per day	First visit of day	Last visit of day
		(n per sow and day)	(n per sow)	(hour)	(hour)
**Gilts**	4	0	2.8 ± 1.2	8.4 ± 5.1	19.1 ± 4.3
**1**	3	0	3.7 ± 1.2	10.2 ± 3.0	19.9 ± 0.5
**2**	2	0.5	4.5 ± 0.7	5.6 ± 4.0	20.2 ± 2,9
**3**	4	0	5.3 ± 1.0	5.9 ± 2.3	18.3 ± 2.4
**4–6**	5	0.2	4.0 ± 2.5	5.1 ± 2.3	14.4 ± 4.4
**7**	4	0.25	4.0 ± 0.8	3.5 ± 1.1	17.2 ± 1.6
**8–12**	3	0.33	3.7 ± 2.1	3.9 ± 0.7	14.5 ± 4.0
**TOTAL**	**25**	**0.2 ± 0.2**	**4.0 ± 1,6**	**6.0 ± 3.4**	**17.3 ± 3.6**

*The feeding day began at 03.00 am. The too early visits represent visits from 00.00 to 02.30, i.e. expected unawarded visits without access to food

### Activities of the staff

During the days of filming, the staff spent 32 ± 12 minutes within the sow facilities (49 minutes during spring, 31 during summer, 24 during autumn and 23 during winter, respectively). One remark made by the staff was that handling of sows was simplified because the ESF system enabled automagical directing of sows for handling to the collection area when leaving the feeding cubicles.

## Discussion

Undesired behaviour decreases the well-being of loose housed sows, and the incidence of aggressions are influenced by housing system designs, stocking densities, feeding methods and strategies of mixing sows [26]. Indeed, inter-sow aggressions due to the feed restriction during gestation is a major welfare problem in group housing systems that also can affect productivity [27]. Sows may be fed collectively on the floor or in troughs where low ranked sows are unprotected from feed stealers [26]. Low ranked sows can however be protected from feed stealers when fed individually in feeding cubicles, which also will decrease aggression and competition between sows [28, 29]. Still, supportive feeding of sows in need of that remains complicated since sows alter between cubicles when eating. In contrast, transponder controlled ESF systems with exits from the feeding cubicles separated from the entrances have been proven effective in offering individual feeding rations [14, 15].

The ESF system tested offered sows the opportunity to spend time between meals in either a tent with deep-litter straw or a barn with saw dust and chopped straw. It was deemed animal-friendly due to a low level of aggressions (0.05±0.04 interactions head-to-head per sow and day) which was lower than reported from other systems [30, 31]. The system was also beneficial for the staff as sows to be handled automatically could be directed to the collection area when leaving the feeding cubicles.

The sows used their opportunity to choose where to stay between meals. Despite that the number of sows always was higher in the tent with deep litter straw where sows mainly rested, there were always sows in the barn during daytime. In the barn, sows were generally in motion or rooting. As previously has been shown [29, 32], the feeding stations where the exits from the eating cubicles were clearly separated from the entrances secured safe eating also for low ranked sows and the ESF system enabled supportive feeding of sows in need of that.

With the aim to identify returners to heat, the sows passed a pen with a boar in the exit area from the feeding stations. However, as most sows were pregnant and thereby not in heat, there were few interactions with the boar. Instead, the sows used this area mainly for transport to the tent or to the barn. Consequently, there were practically no interactions between sows in this area. Nor were there in principle any interactions between sows in the tent where 60 to 85% of the sows were resting or sleeping, or in the barn where 60 to 90% of the registered behaviours included activities. The area of 254 m^2^ in the barn was established in an already existing building. Considering the lower density of sows there, especially during the winter, it was concluded that the area could be reduced with at least 30% without jeopardizing the functionality of the barn.

As also shown by others [33, 34], the behaviour of the sows varied with the seasons, and the activity level of the sows was lowest during the summer. In principle all interactions between sows took place at the pre-eating area and were most common during winter. During winter, the sows crowded when resting, presumably with the aim to maintain body warmth. They also moved crowded in groups, probably also with the aim to remain warm. This resulted in crowding at the pre-eating area with increased number of sows waiting for access to the feeding cubicles and an increased number of interactions between sows during winter, of which in principle all took place at the entrance to the feeding stations or at the water throughs located there. As sows did not spend less time drinking during winter than during the other seasons, the increased queuing at the water throughs in the pre-eating area during winter was somewhat confusing and might solely have reflected the movement pattern of the sows - resulting in many sows at the pre-eating area of 50 m^2^. Indeed, 71% of the interactions between sows during winter were directed to body or rear, rather indicating interactions between sows due to throng than to ranking interactions that generally are directed to the head of the antagonist [35, 36]. During winter, no interactions between sows took place at the post-eating area and no sow was waiting at the water throughs there, instead sows appeared to prioritise a rapid transfer to the tent and the warm deep litter straw bed there. Thus, the study confirmed that gestating sows can be housed in uninsulated buildings during winter provided that the feeding ration is adapted to the temperature and that they can protect themselves from wind and chill, e.g. by resting crowded and by bivouacking themselves in deep litter straw, as previously have been shown by others [37].

As mentioned, the behaviour of the sows varied with the seasons. During summer, sows were less active, and they rested solitary which indicated an aim to reduce body heat. The decreased activity was also recorded at the feeding stations which resulted in a lower crowding of sows at the pre-eating area, especially at 03.00 when the feeding day started. Instead, the strain on the pre-eating area was highest in the afternoon during the summer. The number of interactions between sows was higher than in spring and autumn, but still 50% lower than during winter. Again, most of the interactions took place at the pre-eating area, but contrasting to the other seasons 15% of the interactions took place at the post-eating area. Around 50% of the interactions in summertime were directed to the head of the antagonist compared to 29% during winter, which possibly indicated that aggressions due to ranking were more common during summer than during the other seasons, including winter.

From a climatic point of view, spring and autumn were most alike, and the indoor temperatures only differed with 3–4 °C between these seasons. Indeed, the behaviours of the sows were most similar during spring and autumn, and the number of interactions between sows were significantly lower than during both summer and winter although the total number of interactions were higher during winter than during summer. Also, the number of aggressions defined as interactions head-to-head was significantly higher during summer and winter than during spring and autumn, but did not differ between summer and winter. The general impression when also taking other days than those included in the study into account was that sows preferred to stay in the barn during warmth and in the tent during chill, as also mirrored by resting individually during summer and in groups during winter.

The restricted feed rations during gestation lead to a constant motivation to eat which increase the risk of aggressions due to increased competition [28], and as pig herds grow larger it is almost impossible for the farmers to assess every individual animal and assure its wellbeing [38]. The ESF system used in this study allowed individually adapted feeding of (thin) sows in need, but the system never triggered the alarm for sows with decreasing weight. The two residential options between meals ensured a high welfare in terms of absence of stereotypical behaviours and low incidences of aggressions, and as sows visited the feeding cubicles around five times a day a certain amount motion was ensured among the sows. The merged effect of welfare and motion was mirrored by lower incidences of medical treatments and culling of sows than the national mean [24, 25]. Also, the reasons for replacement of sows differed from the PigVision mean [25], which resulted in a more strategic genetic selection of breeding stock within the ESF system with varied residential options for gestating sows.

Indeed, the interest in ESF systems for group-housed gestating sows is growing since ESF systems offer benefits, such as individually adjusted feeding through transponder-identification of sows and efficient use of space for large groups because each feeding cubicle serves many sows. However, ESF systems also have challenges. The first generation of ESF systems combined entries and exits to the feeding cubicles in a blind alley which contributed to vulva biting and aggressions [39]. Thus, to exploit the potential of ESF systems, additional measures to protect particularly low-ranked (often younger) sows who tend to suffer more injuries and rank lower in the feeding order [40, 41] were needed and have also been developed [10, 36]. As shown in this study, enough numbers of and correctly designed feeding stations that obstruct feed stealing and sufficient space for low ranked sows to evade interactions with high ranked sows between meals can protect low ranked sows and prevent aggressions.

Concurring a previous report [31] there was a high activity among the feeding cubicles when the feeding day started at 03.00 during autumn, winter and spring, but not during summer when sows appeared to use the relatively cool morning for relaxing. Despite the few aggressions between sows, they were eating in a strict hierarchical order. Old sows ate first and gilts last, as previously also have been described by others [31], and this order was maintained also during late stages of pregnancy (four days before expected farrowing). Sows from the third to the sixth parity generally wean more piglets than older sows [42] as also shown in this study, but they still were inferior to elder sows in hierarchy – and superior to younger sows.

The deep litter straw in the tent was appreciated by the sows, and straw has been the most common enrichment and bedding material for group housed sows [43]. Straw has also been concluded to improve natural behaviour and welfare [44]. The consumer perception is that animals raised with straw have better welfare [45] and experts on pig welfare assign considerable importance to the availability of straw substrates in their welfare assessment of housing systems [4]. However, straw has also been associated with disadvantages, mainly relating to cost, increased labour requirements, hygiene concerns and, most importantly, incompatibility with manure and drainage systems. [45]. Further, straw has previously been concluded not effective in reducing fighting in newly mixed groups [46, 47], although straw reduced injuries and lameness [47]. However, these studies focused on straw solely and did not consider other factors, such as extra space or alternative housing designs, which in combination with straw reduced aggressiveness [30, 48], as also shown in this study.

Due to the large total space per sow, the system scrutinised may be dismissed for financial reasons. It must however be considered that the buildings tested were cheap as they were uninsulated and either built with thinly sliced boards or raised as a permanent tent, which justified the system from an economic point of view. Still, as the buildings were uninsulated, sows needed protection in terms of shelter from wind and chill, which was achieved by solid pen walls in the barn and deep litter straw in the tent. As deep litter beds are established upon solid floors, they do not interfere with any manure draining system.

## Conclusions

The system with varied residential options between meals for gestating sows and an individually adapted electronic feeding system ensured motion of sows because they visited the feeding stations around five times a day. The system was concluded as animal-friendly since it involved few aggressive incidents, no stereotypic behaviours, few medical treatments, and few gestating sow culls. The sows utilised their ability to eat individually and to choose where to stay between meals, and the enriched environment enhanced their overall well-being without reducing productivity. The study also confirmed previous experiences that gestating sows can be housed in and perform well in uninsulated buildings during winter, provided that the feeding rations are adapted to the temperature and that sows can protect themselves from wind and chill, e.g. by resting crowded and by bivouacking themselves in deep litter straw.

## Data Availability

No datasets were generated or analysed during the current study.

## Abbreviations

ESF: Electronic sow feeding
PPDS: Post Partum Dysgalactia Syndrome
SVA: Swedish Veterinary Agency
THI: Temperature-Humidity Index
